# A survey of few-shot learning in smart agriculture: developments, applications, and challenges

**DOI:** 10.1186/s13007-022-00866-2

**Published:** 2022-03-05

**Authors:** Jiachen Yang, Xiaolan Guo, Yang Li, Francesco Marinello, Sezai Ercisli, Zhuo Zhang

**Affiliations:** 1 School of Electrical and Information Engineering, Tianjin University, Tianjin, China; 2 College of Mechanical and Electrical Engineering, Shihezi University, Xinjiang, China; 3 Department of Land Environment Agriculture and Forestry, University of Padova, Legnaro, Italy; 4 Department of Horticulture, Faculty of Agriculture, Ataturk University, Erzurum, Turkey

**Keywords:** Few-shot learning, Deep learning, Data augmentation, Metric learning

## Abstract

With the rise of artificial intelligence, deep learning is gradually applied to the field of agriculture and plant science. However, the excellent performance of deep learning needs to be established on massive numbers of samples. In the field of plant science and biology, it is not easy to obtain a large amount of labeled data. The emergence of few-shot learning solves this problem. It imitates the ability of humans’ rapid learning and can learn a new task with only a small number of labeled samples, which greatly reduces the time cost and financial resources. At present, the advanced few-shot learning methods are mainly divided into four categories based on: data augmentation, metric learning, external memory, and parameter optimization, solving the over-fitting problem from different viewpoints. This review comprehensively expounds on few-shot learning in smart agriculture, introduces the definition of few-shot learning, four kinds of learning methods, the publicly available datasets for few-shot learning, various applications in smart agriculture, and the challenges in smart agriculture in future development.

## Background

Deep learning is a new direction in the field of machine learning. Its ultimate goal is to make machines have the ability of analysis and learning like human beings, and can recognize characters, images, sounds and other data. Among them, convolutional neural network (CNN) is the most mainstream model. A complete deep learning process is divided into two stages: training and testing. For a specific task, in the training strategy, the labeled training set, i.e., training data, is used to adjust the parameters of CNN for hundreds of rounds, then a trained model is obtained. In the test strategy, the test set, i.e., test data, is used to evaluate the performance of the model.

In the image recognition competition ILSVRC held in 2012, AlexNet [1] won the championship by far surpassing the second place, the power of deep learning was finally shown in front of the world. With the continuous improvement of deep learning technology and hardware capabilities, artificial intelligence has developed more and more rapidly, and remarkable achievements have been made in many fields such as smart agriculture [2–5], medical treatment, finance, driverless, and so on [6–12]. Nowadays, more and more scholars begin to pay attention to how to apply in deep learning in the field of smart agriculture. For example, the classification network is used to automatically identify pests [13], weeds [14], plant diseases [15], the detection network is used to detect pests [16], and for plant breeding [17]. The introduction of artificial intelligence makes agriculture more intelligent and automatic, and effectively reduces economic losses.

**Fig. 1:**
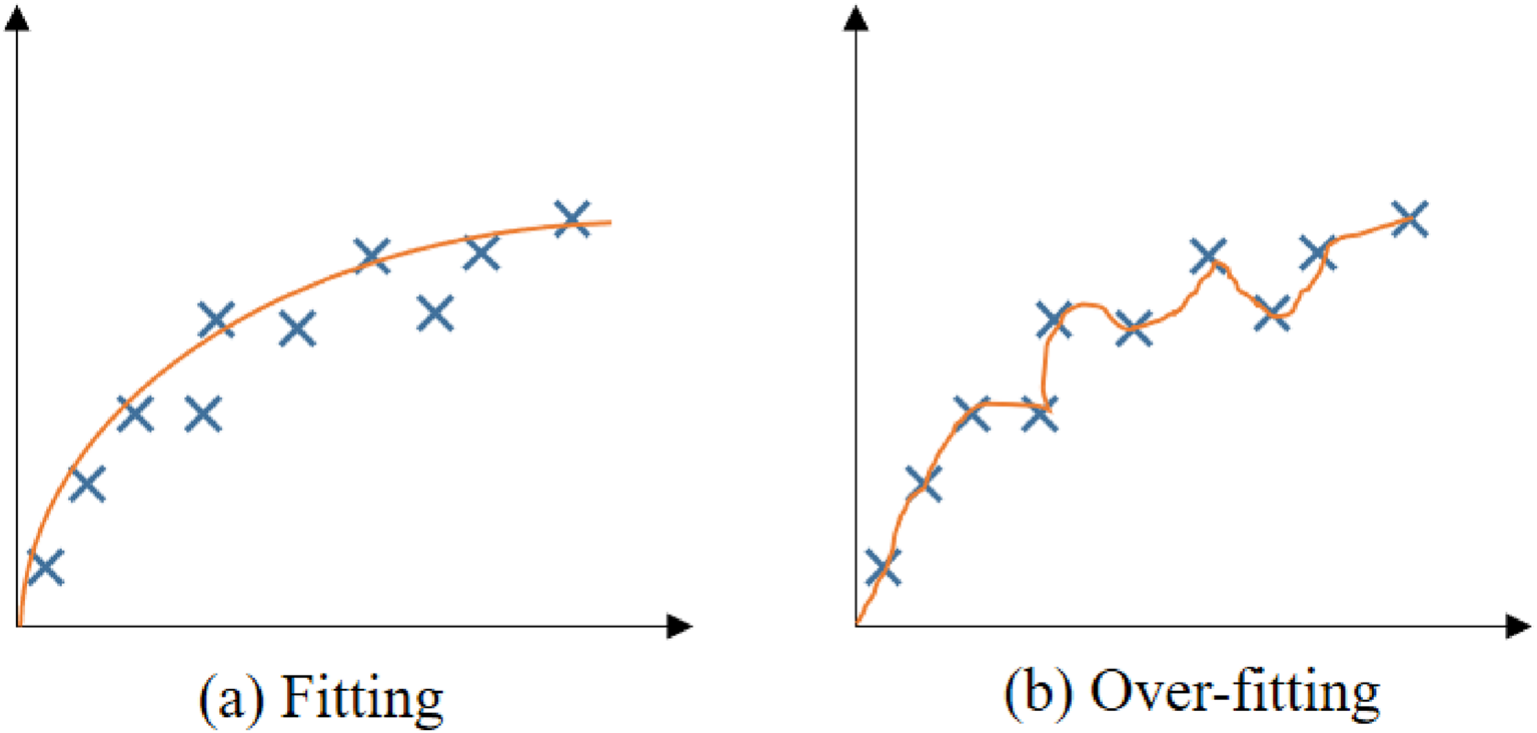
Fitting and over-fitting curves

With the more and more extensive use of deep learning, its shortcomings are gradually exposed, that is, an excellent model relies heavily on massive amounts of training data. However, in the field of agriculture, the number of samples we can obtain is often very limited since data acquisition involves security, ethics, resources, and cost. Generally, when training large models based on a small number of samples, there will usually be a serious over-fitting problem, i.e., the network parameters are over-fitted, and the accuracy is very high in training strategy and low in testing strategy. The difference between over fitting and fitting is shown in Fig. 1. In this case, the traditional deep learning algorithm loses the almost magical performance. However, humans only need to see a new thing once to recognize it. So, researchers imitate the fact that human beings can learn quickly then propose few-shot learning. Different from the traditional networks, the few-shot learning model can train a classifier with a good performance by inputting only one or a few labeled images.

Now there have been many studies on agricultural few-shot learning, whose models can successfully identify crop pests [18], plant disease [19], plant breeding [20] and so on. The emergence of few-shot learning has successfully brought AI into the era of few-shot. The deep learning tasks no longer depend on massive datasets, which greatly reduces the difficulty and cost of obtaining training data in many fields, including plant biology.

### Bibliometrics

**Fig. 2:**
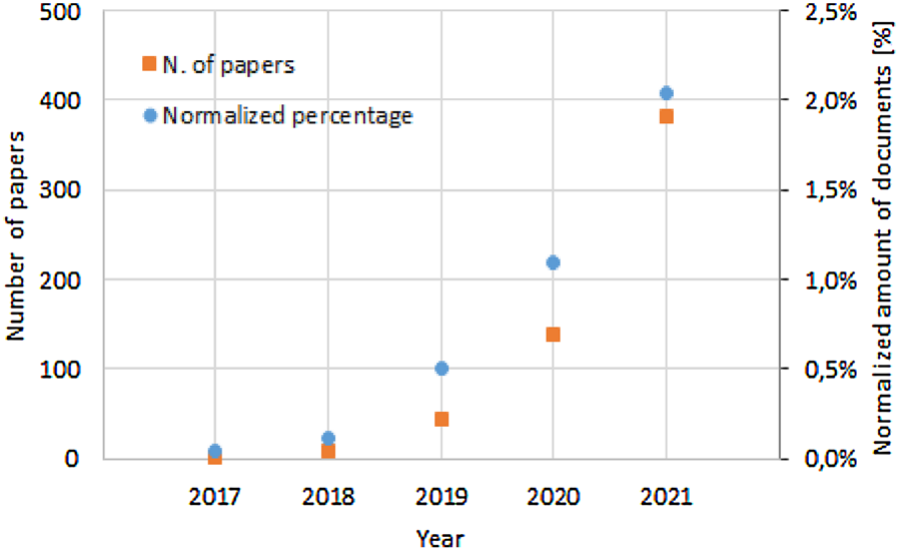
Actual number and normalized percentage of publications related to the few-shot learning topic in the last 5 years

Before further proceeding with analysis and classification of few-shot learning, a systematic quantitative review was carried out, based on documents indexed by the Scopus database. The advanced search tool was implemented to extract pertinent documents indexed by Scopus with the following query: "(TITLE-ABS-KEY ("few shot") AND (TITLE-ABS-KEY (artificial AND intelligence) OR TITLE-ABS-KEY (learning) OR TITLE-ABS-KEY (neural AND network) OR TITLE-ABS-KEY (classification))) AND (LIMIT-TO (DOCTYPE, "ar") OR LIMIT-TO (DOCTYPE, "re")) AND (LIMIT-TO (LANGUAGE, "English"))". A total of 595 documents was reported, shown in Fig. 2, with a clear increase from the two works in 2017 (0.04% if normalized against the papers in the field of “Artificial Intelligence”), 44 in 2019 (0.50% normalized value) and 389 in 2021 (2.04% normalized value). The trend of recent literature publications reflects the vigorous development and widespread interest in this new technology (few-shot learning).

**Fig. 3:**
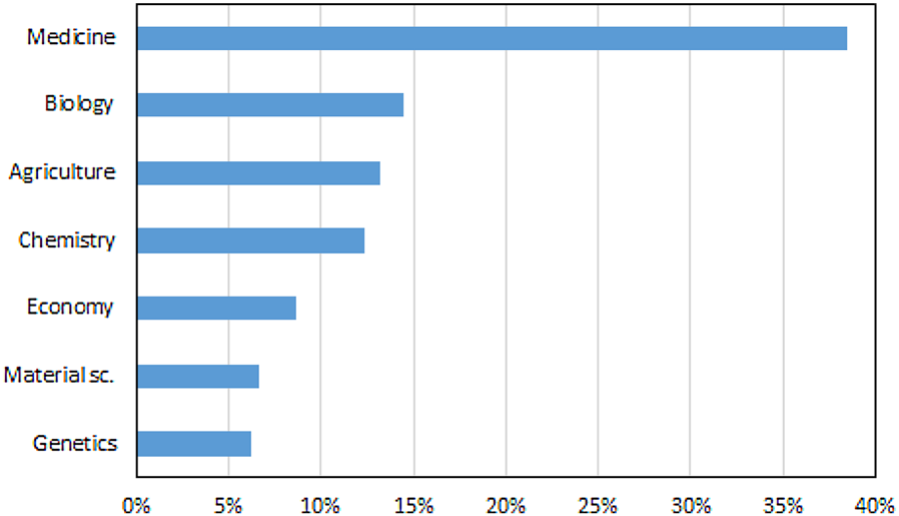
Field of interest of applied sciences documents

The 595 documents were analyzed using text mining tools, taking advantage of the frequency functions available within Microsoft Excel. Terms appearing in title, keywords, and abstract were extracted in order to quantify and study occurrences. The large majority of documents (58%) discuss purely theoretical approaches, from the informatics, mathematical or statistical point of view. But nowadays, few-shot learning is in the stage of rapid development, and few-shot learning technology has been used in many fields [18, 21–25]. Indeed, the remaining documents (about 250) have a more applicative scope, mainly focused on medicine (38%), biology and agriculture (27%), chemistry (12%), and other fields, shown in Fig. 3.

### Main categories

**Fig. 4:**
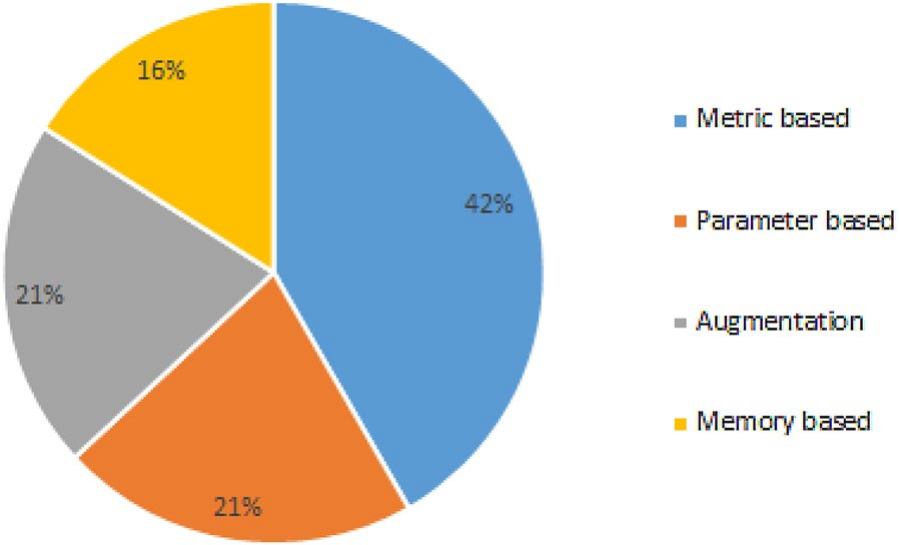
Partition of scientific papers based on applied approach

The existing few-shot learning methods are roughly divided into four categories: methods based on data augmentation, methods based on metric learning, methods based on external memory, and methods based on parameter optimization, homogeneously rationed between scientific documents, shown in Fig. 4.

The method based on data augmentation solves the over-fitting problem by expanding the number of samples by reusing the originals, the method based on metric learning classifies samples by simulating the similarity between samples, the method based on external memory helps model learning by adding additional storage and memory modules, and the method based on parameter optimization solves the problem by learning how to optimize parameters.

The specific contributions of this survey are as follows:

1. Elaborate the definition of few-shot learning, systematically sort out the methods of few-shot learning according to four different strategies, and list the main networks of various methods.
2. Systematically sort out the datasets currently used for few-shot learning, and introduce them in detail. The performance of mainstream methods based on four different strategies on the benchmark set is listed.
3. Introduction of the applications of few-shot learning in smart agriculture in detail, and list the existing challenges in smart agriculture few-shot learning.

## Overview

Different from the traditional machine learning and deep learning methods, few-shot learning has its special learning methods and training strategies. In this section, we will introduce the theoretical definition, symbolic definition, and specific training strategy of few-shot learning in detail.

### Definition

Given a specific task $\Gamma$, which contains a small amount of available dataset ${D}_{T}$ with supervision information and auxiliary dataset ${D}_{A}$ not related to $\Gamma$, the goal is to build a function $f$ for task $\Gamma$. The completion of the task uses little supervision information in ${D}_{T}$ and knowledge in ${D}_{A}$, and finally maps the input to the target task.

### Symbolic definition

Few-shot learning is generally regarded as an *N*-way *k*-shot problem. *N* refers to the number of categories contained in each task support set, *k* refers to the number of samples in each category, and generally *k* does not exceed 20. The sample category is represented by ${C}_{i}$, and the training set and test set are represented by ${D}_{T}$ and ${D}_{A}$ respectively.

### Training strategy

**Fig. 5:**
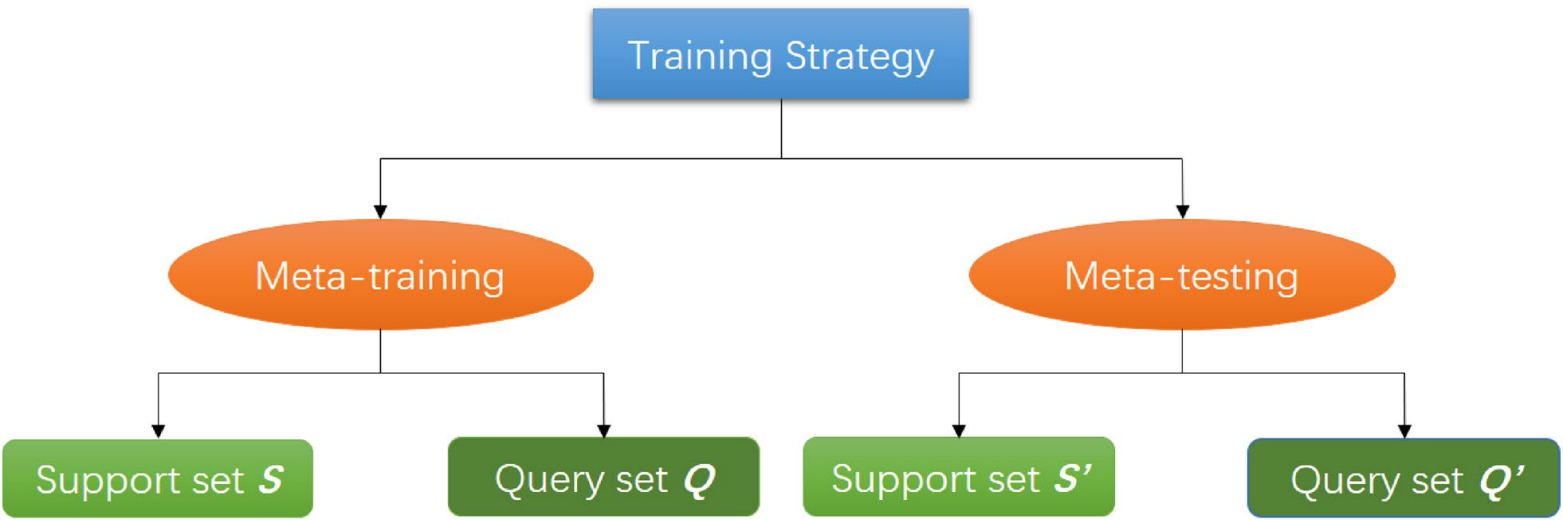
The training strategy of few-shot learning

The general deep learning process only includes training, validation, and testing, but due to the particularity of few-shot learning, its training and testing are very different from deep learning. As shown in Fig. 5, the training strategy includes meta-training and meta-testing in few-shot learning. In these two stages, they have their support set *S* and query set *Q*. Support set can be understood as training set during meta-training, query set can be understood as test set during meta-training, and *S’* and *Q’* in meta-testing are used to fine-tune and finally test the performance of the network respectively.

## Methods

Nowadays, few-shot learning is in the stage of rapid development. At present, the popular few-shot learning methods can be roughly divided into four categories according to strategies, namely, methods based on data augmentation, methods based on metric learning, methods based on external memory, and methods based on parameter optimization.

### Method based on data augmentation

**Fig. 6:**
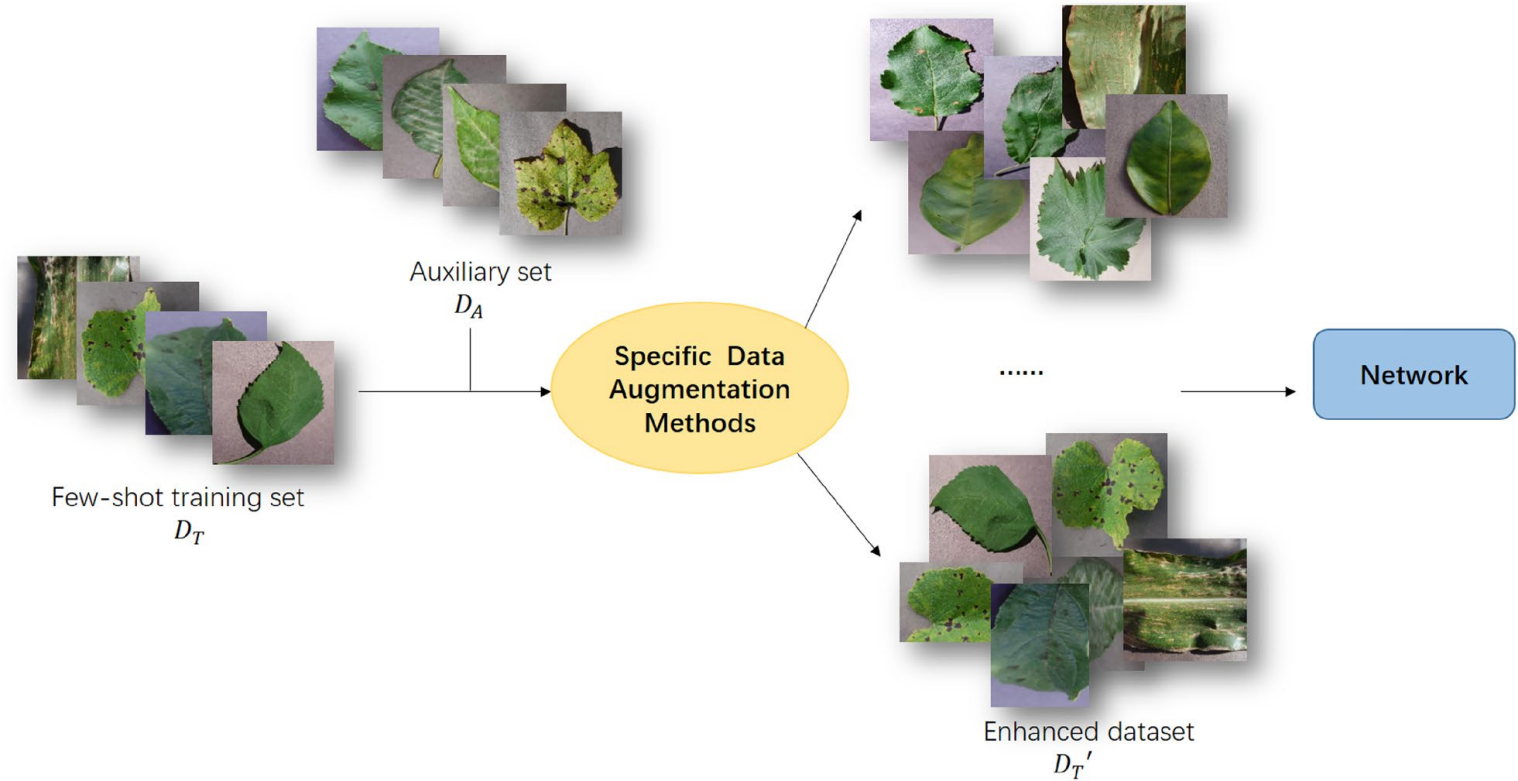
The methods based on data augmentation

The characteristic of few-shot learning is the lack of labeled samples. To solve this problem, the simplest and most direct method is to expand the number of samples. Data augmentation refers to the use of specific methods to generate new samples from the original samples with the same distribution according to the original samples when there are a small number of samples, to achieve the purpose of expanding the dataset. The workflow is shown in Fig. 6. When the number of samples is expanded to the number of samples required by traditional deep learning, the over-fitting problem faced by few-shot learning is naturally solved. The formula for sample generation is as follows:

1 $${D}_{T}^{^{\prime}}=f\left({x}_{i}\right),{y}_{i}$$

where $\left({x}_{i},{y}_{i}\right)$ represents the original image, f represents the generation function.

According to the early data augmentation method, the original sample is operated on by using fixed rules such as rotation, translation, clipping, and flipping. Although these methods are simple, they can effectively expand the amount of information of the sample, because for the neural network, even a rotation will be regarded as a new sample. However, the traditional method has its limitations. For example, for a non-square sample, the rotation will lead to the loss of the size information of the original image, and the amount of information of these generated images is limited. For today's more intelligent image augmentation technology, the most representative is the Generative Adversarial Network (GAN) [26]. GAN includes two different networks: generator and discriminator. The task of generator is to generate examples that look close to the real and similar to the original data through random noise. The task of discriminator is to judge whether a given instance is true or forged. Li et al. [27] solved the problem of lack of discrimination ability and diversity of generated samples by generating samples with conditional Wasserstein Generative Adversarial Networks (cWGAN) and adding classification regularizer and anti-collapse regularizer. Similarly, Eli Schwartz et al. used an automatic encoder [28] to find the deformation between different samples of the same category, then used it to generate new samples for other category samples, and finally used the expanded dataset to train the classifier. The characteristic of these methods is to find a generation function based on the existing sample knowledge to generate amplified samples. Amit Alfassy et al. Creatively started with sample labels [29], used the intersection, union, difference, and other relationships between label sets, learned implicit semantic information from images, enhanced the dataset, expanded the feature information contained in the feature space, and solved the problem of multi-label classification of few samples.

### Method based on metric learning

**Fig. 7:**
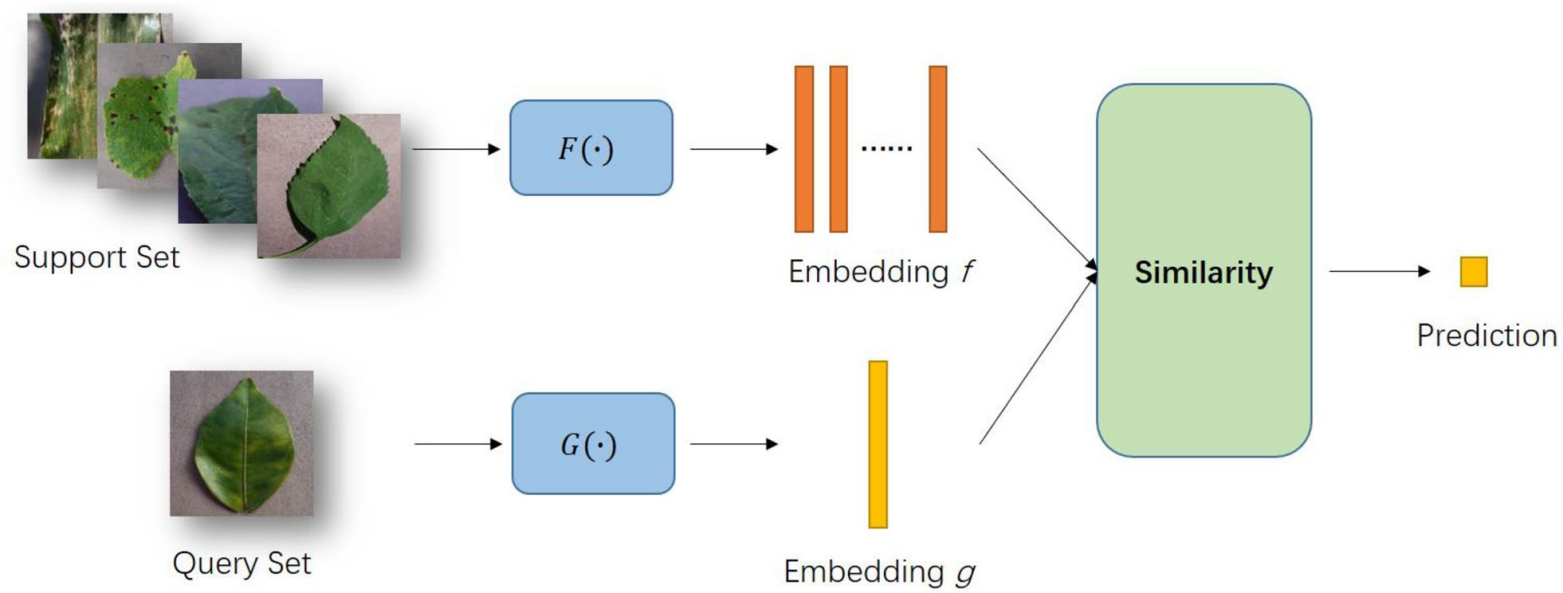
The methods based on metric learning

The few-shot learning method based on metric learning aims to measure the distance between support set samples and query set samples through a specified or learnable metric method, to complete the task of few-shot classification. The performance of this method depends on the measurement method. An effective measurement method can make the network get rid of the over-fitting problem caused by too few samples and too deep a network structure. The workflow is shown in Fig. 7. The formula is as follows:

2 $$p=F(d\left({x}_{i},{x}_{j}\right),\theta )$$

where d represents the distance function, $\theta$ represents the network’s parameters.

The earliest measurement method is the Siamese network [30] proposed by Gregory Koch in 2015. It uses the same weight-sharing network model to extract the features of two different images. If the distance between the two features is very close, they are considered to be the same category. Specifically, after the features of the two images are obtained, the absolute value of the difference between the two features is calculated by L1 distance, multiplied by a weight, and then all the feature values are added to obtain the distance score between (0,1) through sigmoid function. The idea of MatchingNet [31] proposed by Oriol Vinyas et al. is very similar to that of the Siamese network. The difference is that the MatchingNet adopts the long short-term (LSTM) network integrating attention mechanism to extract features, and cosine distance is used to measure the gap between features. Prototype network [32] is an upgraded learning method proposed by Snell and colleagues based on MatchingNet. This method calculates the average value of high-dimensional features of each class of sample as the prototype of this class, and calculates the Euclidean distance between the test sample and each class of prototype, to predict the label of this class of sample. There are still many methods based on fixed measurement methods like these, such as using earth mover's distance [33] proposed by Zhang et al. to measure the distance between support set and query set samples. In addition to the measurement method of fixed rules, Sung et al. creatively proposed using the RelationNet to learn the measurement method [34]. The model has two modules, the first is an embedded module for extracting high-dimensional feature information, and the second is a correlation module for stitching the features of the query set image and the features of each image in the support set. The similarity score is calculated between the two images to judge whether the two images are from the same category. After that, Zhang et al. proposed an upgraded version of RelationNet2 [35]. This network uses SENet to replace the original conv-4 structure. Different from the original vision, which only calculates the relationship score for the last layer, the network calculates the relationship score in the middle stage of extracting the network. In addition, the feature map extracted by the network is the mean and variance. Finally, a new feature map is reconstructed by the re-parameterization method.

### Method based on external memory

**Fig. 8:**
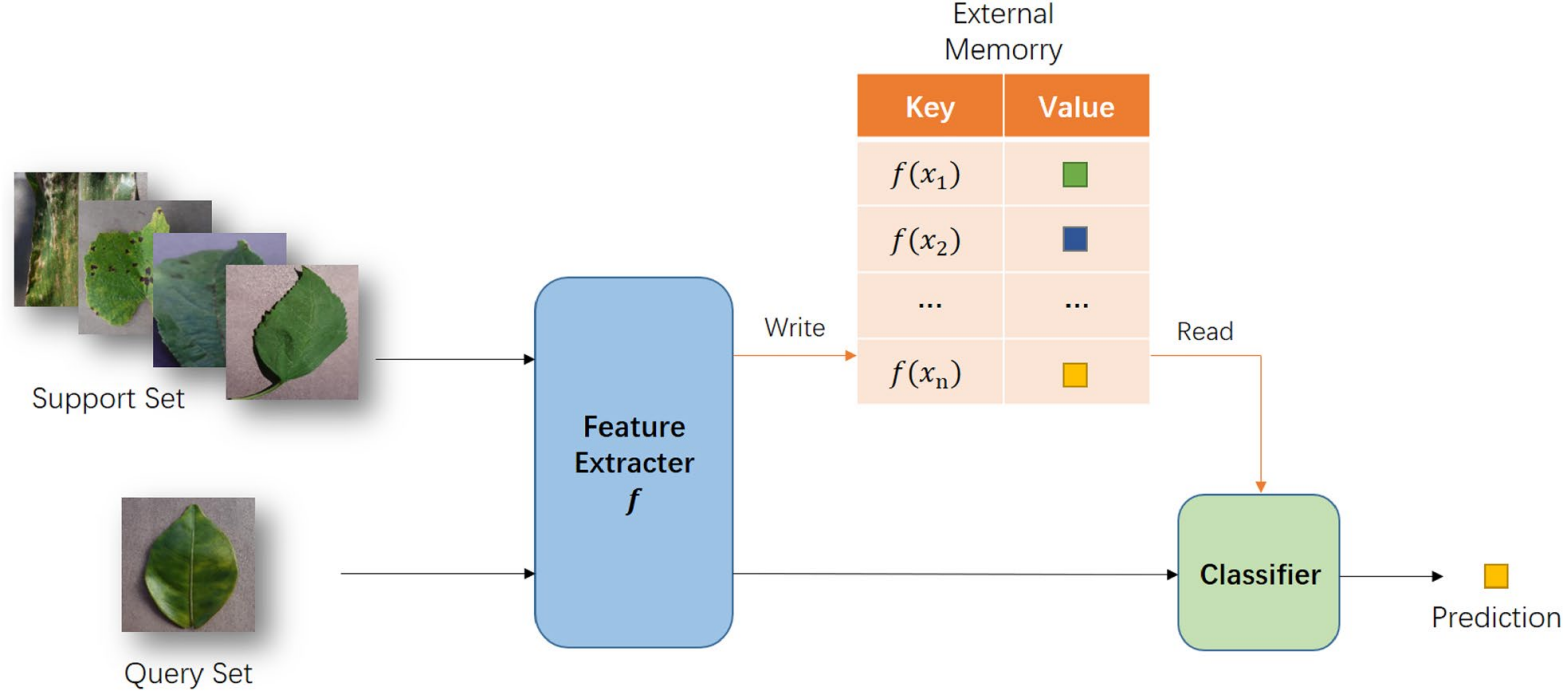
The methods based on external memory

We know that the long short-term memory (LSTM) [36] can remember long-term memory (data entered earlier) and short-term memory (data entered currently) at the same time. The few-shot learning based on external memory imitates this feature. Additional memory modules are added to the model to remember the characteristic information of a few samples, to complete the few-shot learning task. The workflow is shown in Fig. 8. The formula of the memory module is as follows:

3 $$M\left(i\right)=({M}_{key}\left(i\right),{M}_{value}\left(i\right))$$

Santoro and colleagues first proposed the idea of using external memory to solve few-shot learning tasks in 2016. Their proposed memory enhanced neural network (MANN) [37] can solve the disadvantage of LSTM instability, learn from the idea of the neural Turing machine (NTM), and adopt the method of external memory to quickly learn the information contained in samples. Because NTM can not only update the weight slowly to achieve long-term storage, but also short-term storage through external memory modules, NTM is very suitable for meta-learning and few-shot classification problems. MetaNet [38] proposed by Munkhdalai et al. adopts a few-shot learning method combining meta-learning and external memory. MetaNet is composed of two main learning units: base-learner and meta-learner, and has an external memory module. The idea of memory matching networks [39] proposed by Qi Cai et al. is relatively simple and easy to understand. The network first extracts the features supporting the centralized pictures, and stores them in the memory module together with the corresponding category labels to form a "key-value pair", and then extracts the features of the query set pictures and compares them with the feature information read from the memory module, select the category with the highest similarity as the category of query pictures. Kaiser et al. proposed the lifelong memory module [40]. During training, the network saves the characteristic information of each category sample and the corresponding label value. During testing, the nearest neighbor idea is used to select the k samples closest to the query sample, and then predict the label of the sample.

### Method based on parameter optimization

**Fig. 9:**
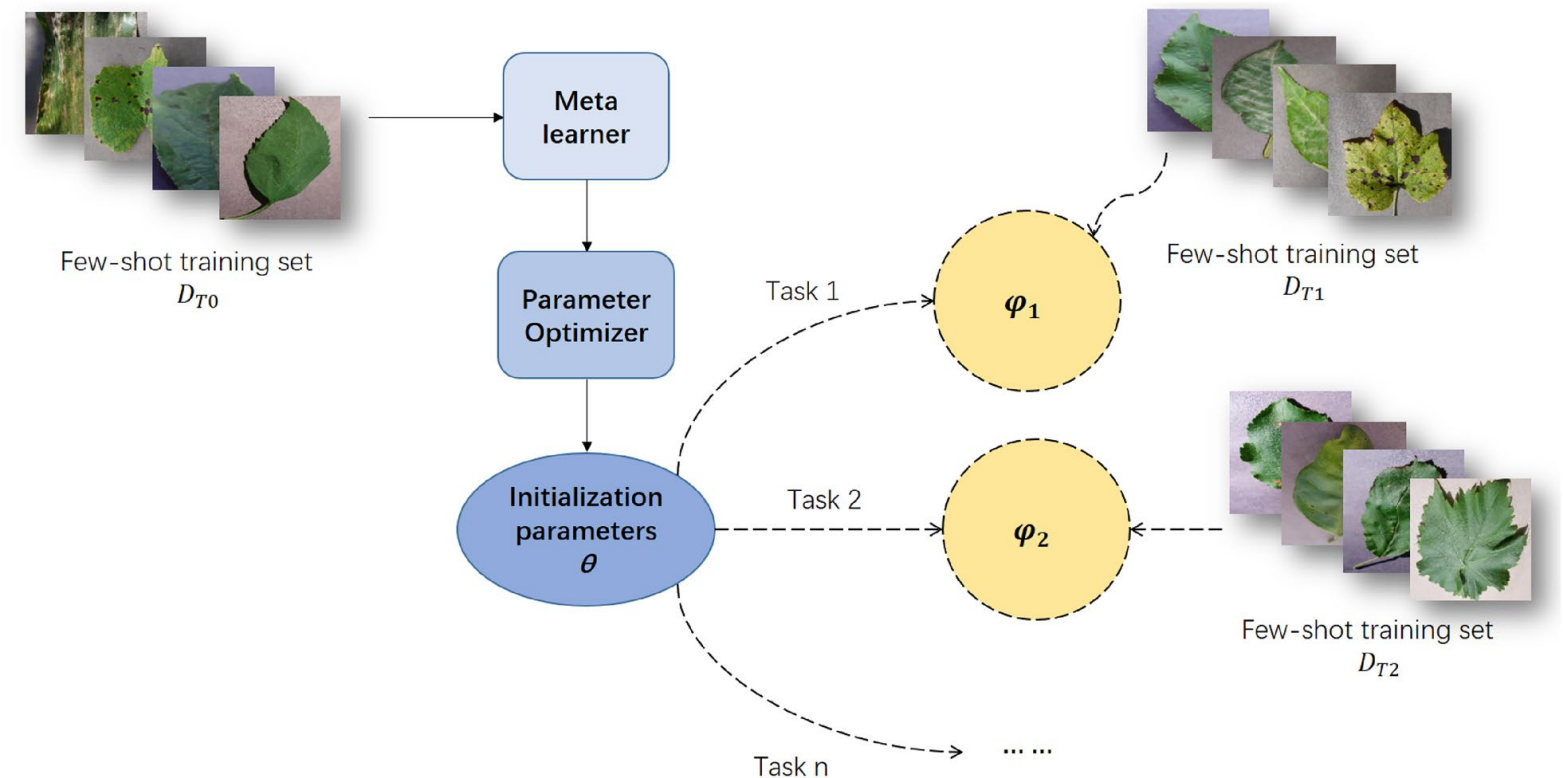
The methods based on parameters optimization

Traditional deep learning essentially uses a large number of samples to optimize the parameters of the network model. Through the gradient back propagation of the network, the parameters are fitted to the optimal value. However, when the number of samples is small, the deep model will be over-fitted. The few-shot learning method based on parameter optimization is to learn how to optimize parameters through an optimizer, to solve the problem of network over-fitting. The workflow is shown in Fig. 9. The formula of network prediction is as follows:

4 $$p=F(f({x}_{i}),w(\theta ))$$

where $f\left(\bullet \right)$ represents the feature extracting function, $w(\theta )$ represents the function that learns optimizer parameters, $F\left(\bullet \right)$ represents the function that finally gets the classification result.

The most classic optimization-based method is MAML [41] proposed by Finn et al. The general neural network usually uses random initialization parameters, or uses pre-trained initialization parameters, and then updates the parameters through the gradient. However, MAML aims to learn an appropriate initialization parameter θ. In the face of new tasks, only a few steps of gradient updating can have a good effect. Therefore, MAML can achieve "model-agnostic", which is suitable for few-shot regression, image classification, and even reinforcement learning. Jamal and others believe that even learning an initialization parameter will have a preference for the training set, resulting in the decline of generalization ability. Based on the maximization of entropy reduction and the minimization of inequality, the authors designed a "task-agnostic" TAML [42] to solve the problem of training preference. Based on MAML, Nichol et al. Proposed the algorithm Reptile [43], which is directly initialized with the parameters with the most gradient of vector difference. This algorithm is much simpler than MAML, but it is mathematically equivalent to the first-order approximate MAML. Elsken et al. introduced neural architecture search (NAS) into few-shot learning, combined DARTS [44] with Reptile and proposed MetaNAS [45]. The network should learn not only the initialization parameters, but also the network structure. Guo et al. proposed a few-shot classification method for weight generation [46], which combines query set image and support set image through mutual information (MI) and attention mechanism to jointly generate the weight of classifier.

## Few-shot datasets and performance of different methods

As few-shot learning is studied more and more deeply, the publicly available benchmark datasets dedicated to few-shot learning are also increasing. This section introduces several few-shot datasets in detail, shown in Table 1. The performance of the few-shot learning methods introduced above on the three benchmark sets of Omniglot, mini-ImageNet, and tiered-ImageNet are summarized in Table 2.

**Table 1:** Various few-shot datasets

Dataset	Source	Number of classes	Number of images	Image Size
Omniglot CUB mini-ImageNet tiered-ImageNet Fewshot-CIFAR100 CIFAR-FS	– – ImageNet ImageNet CIFAR100 CIFAR100	1623 200 100 608 100 100	32,460 11,788 60,000 779,165 60,000 60,000	28 × 28 84 × 84 84 × 84 84 × 84 32 × 32 32 × 32

**Table 2:** Performance of different methods on benchmarks

Method	Model	Backbone	Omniglot		Mini-ImageNet		Tiered-ImageNet	
			1-shot	5-shot	1-shot	5-shot	1-shot	5-shot
Data Augmentation	AFHN [27]	ResNet-18	–	–	62.38 ± 0.72	78.16 ± 0.56	–	–
	∆-encoder [28]	ResNet-18	–	–	59.9	69.7	–	–
Metric Learning	MatchingNet [31]	ResNet-12	97.9	98.7	63.08 ± 0.80	75.99 ± 0.60	68.50 ± 0.92	80.60 ± 0.71
	RelationNet [34]	Conv-4	99.6 ± 0.2	99.8 ± 0.1	50.44 ± 0.82	65.32 ± 0.70	54.48 ± 0.93	71.32 ± 0.78
	ProtoNet [32]	ResNet-12	98.8	99.7	60.37 ± 0.83	78.02 ± 0.57	–	–
	DeepEMD [33]	ResNet-12	–	–	65.91 ± 0.82	82.41 ± 0.56	71.16 ± 0.87	86.03 ± 0.58
External Memory	MetaNet [38]	ResNet-12	99.9	–	49.21 ± 0.96	–	–	–
	MMNet [39]	CNN + LSTM	99.28 ± 0.08	99.77 ± 0.1	53.37 ± 0.48	66.97 ± 0.35	–	–
	[40]	ResNet-10	–	–	55.45 ± 0.89%	70.13 ± 0.68%	–	–
Parameter Optimization	MAML [41]	Conv-4	98.7 ± 0.4	99.9 ± 0.1	48.70 ± 1.75	63.11 ± 0.92	–	–
	Reptile [43]	Conv-4	95.39 ± 0.09	98.90 ± 0.1	47.07 ± 0.26%	62.74 ± 0.37%	–	–
	MetaNAS [45]	CNN	–	–	63.1 ± 0.3	79.5 ± 0.2	–	–

### Few-shot datasets

#### Omniglot

**Fig. 10:**
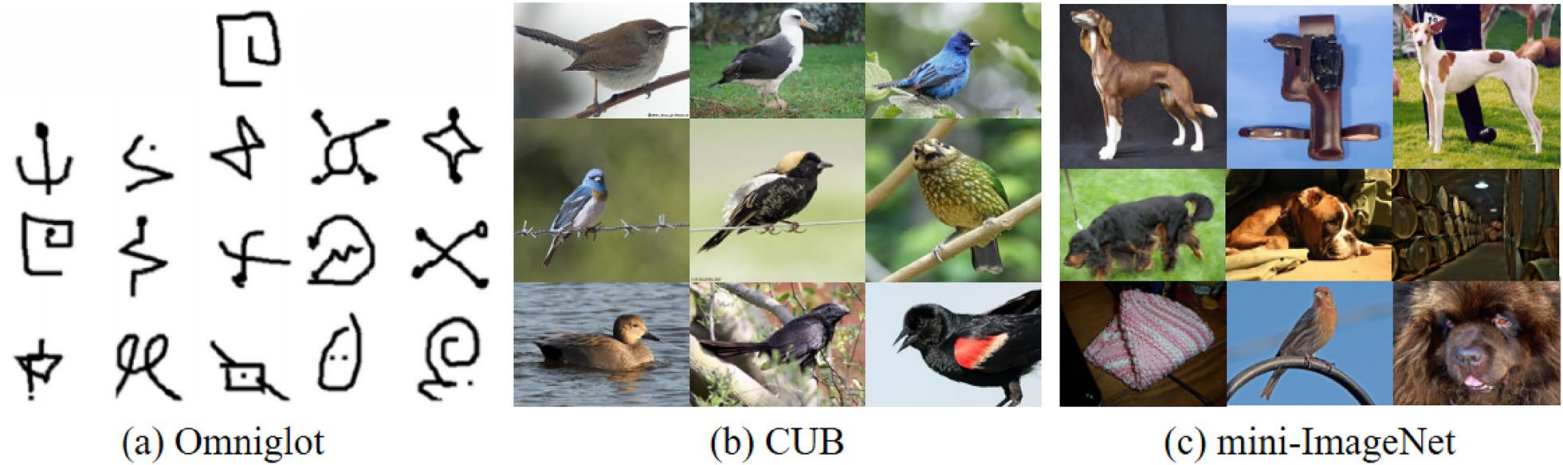
Some images in Omniglot, CUB, and mini-ImageNet

The Omniglot [47] dataset, shown in Fig. 10a, is composed of 1623 categories of different handwritten characters from 50 different letters. Each type of character is handwritten by 20 different people, which is equivalent to that this dataset has 1623 categories, 20 samples of each category, and the size of each picture is 105 × 105. Compared with the 10 categories of the MNIST dataset, in which each category has thousands of samples, the Omniglot dataset is often called MNIST transpose. Each image in Omniglot is paired with stroke data, the coordinate sequence is [x, y, t], and the time *t* is in milliseconds, but we don't often use it in few-shot learning.

#### CUB

CUB200-2011 dataset, shown in Fig. 10b, is a fine-grained dataset proposed by the California Institute of technology in 2010. It is also the benchmark dataset for fine-grained classification and recognition. The dataset has 11,788 bird images and 200 different bird subclasses. Among them, the training set contains about 6000 images, and the test set contains nearly 5800 images. Each image contains bird's bounding box information, image tag information, key part information of birds, attribute information of birds, etc. Although CUB is mainly used in fine-grained classification and recognition, it is also very suitable as a dataset for few-shot learning because of its wide variety and a small number of images in each category.

#### Mini-ImageNet

Mini-Imagenet, shown in Fig. 10c, is derived from the classic dataset ImageNet [48], proposed by the DeepMind team. ImageNet is a very classic large-scale image dataset, which is organized and collected by Stanford Professor Li Feifei and others, including more than 20,000 categories, more than 14 million annotated images, and at least 1 million framed images, with no less than 500 images in each category. ImageNet is widely used in many fields, such as image classification, object location, object detection, and video object detection, which has benefited the majority of researchers and educators. Compared with ImageNet, mini-ImageNet is much smaller. It contains 100 categories, each category has 600 samples, and the size of each image is 84 × 84. It was first used in few-shot learning research by the DeepMind team. There are 64 categories in the training set, 16 categories in the validation set, and 20 categories in the test set. In addition, additional train.csv, val.csv, and test.csv files are required as comments. Mini-ImageNet is more complex than CIFAR-10 and more suitable for experimental research, so it has also become the benchmark dataset for few-shot learning.

#### Tiered-ImageNet

Tiered- ImageNet is also from ImageNet, which was put forward for the first time by Ren et al. [49]. Tiered-ImageNet has 34 categories, which are divided into 20 for training, 6 for verification, and 8 for the test. Each category has 10–30 classes. Such a division method can ensure that all training categories are fully separated from test categories, which is different from mini-ImageNet. At present, as a standard set, this dataset is widely used in the comparison with few-shot learning tasks.

#### Fewshot-CIFAR100

The dataset was first summarized and sorted by Boris N. Oreshkin et al. [50], its content is the same as the original dataset CIFAR-100, with 100 categories, 600 images in each category, and the image size is 32 × 32. To make it suitable for few-shot learning, the author divides these 100 classes into 20 superclasses. The training set has 60 classes, belonging to 20 superclasses, and the verification set and test set have 20 classes, belonging to 5 superclasses respectively.

#### CIFAR-FS

The full name of CIFAR-FS is CIFAR100 Few-Shots, which is the same as Fewshot-CIFAR100 from the CIFAR100 dataset and was first proposed by [51]. It divides CIFAR 100 into training set (64 classes), validation set (16 classes), and test set (20 classes).

### Performance of different methods on benchmarks

We uniformly compare the classification accuracy of the network in the case of 5-way and 5-shot on the three datasets of Omniglot, mini-ImageNet, and tiered-ImageNet. Because the feature of Omniglot is too simple, the new few-shot learning network is no longer verified on this dataset. However, because it is very classic, we still take it as the standard to evaluate the excellence of the network. See Table 2 for specific results.

## Applications

Few-shot learning was initially experimented with in the field of computer vision and achieved initial results in image classification. It does not need to rely on massive samples during training, which reduces the time cost and money cost of obtaining samples, so it is welcomed in many fields like smart agriculture now. This section introduces the various few-shot learning applications in smart agriculture.

### Plant disease recognition

In agricultural and plant properties, plant diseases are one of the key factors affecting yield. When diseases occur, timely finding disease types and making corresponding treatment can effectively reduce economic losses. Traditional plant disease recognition is often judged by experts with their eyes. While using few-shot learning, it can greatly increase the recognition speed, recognition accuracy and efficiency. At present, few-shot learning is also the most widely used in agricultural and plant properties for the identification of plant diseases. For example, Liang et al. Used the few-shot learning method based on metric learning to identify cotton leaf spots [19], Wang et al. proposed multi-mode collaborative representation learning based on disease images and disease texts to solve the problem of vegetable disease identification under complex background [52], Argüeso et al. also used the few-shot model based on metric learning to identify 38 plant diseases in the dataset PlantVillage [53], and Zhong et al. used the conditional adversary automatic encoder (CAAE) to identify citrus golden grape diseases [54]. These studies only use a small number of labeled samples to achieve satisfactory results, so that the identification of plant diseases will no longer rely on expert experience and realize automatic identification in the future.

### Weeds or pests identification

In agriculture, the cost of manual weeding and pest control is high, and chemical weeding and pest control will increase harmful components in crops. Both of them do not meet the development requirements of smart agriculture. Therefore, it is necessary to develop an automatic robot [55] to carry out weeding and pest control in vegetable greenhouse environment. Intelligent identification of weeds and pests is the key technology for developing this kind of robot. To solve this problem, there have been some applications. For example, Li et al. used few-shot learning method to effectively identify cotton pests and applied it in embedded terminals [21], Gui et al. combined few-shot learning with hyperspectral to detect soybean heartworm [56]. As for weed detection, at present, only part of the application of traditional deep learning technology is used [57], which may be a new direction of few-shot learning application in agricultural and plant protection research in the future.

### Crop detection

In agriculture, the detection task is equally important. It should be noted that there are differences between detection and classification (or recognition). Classification is to judge the class of the images. The detection task is to find the location of interested targets in the image and classify them. Therefore, the detection task is much more difficult than the classification task. The detection of crops can effectively perceive the position of crops, soil and so on, and help farmers automatically monitor farmland. Specifically, Zhang et al. used UAV technology and few-shot learning to detect the position of crop seeds [58], Kim et al. used few-shot learning to detect the cultivated soil area in the two-dimensional perspective scene to provide farming path guidance for automatic tractors [59], and Li et al. proposed a Siamese domain transfer network (SDTN) structure to detect corn residues [60]. The application of few-shot learning enables people to control the quality of crops with the least resources.

### Plant phenotyping and breeding

Plant phenotype is the three-dimensional expression in space and time after the interaction between gene and environment. It reflects the physical, physiological and biochemical characteristics of cells, tissues, organs, plants and population structure and functional characteristics. In order to maintain a better character of plants, plant phenotypic screening is often used in plant breeding. At present, there are many studies on plant phenotypic breeding by using deep learning [61, 62], but Karami et al. completed the experiments of counting and positioning corn under the condition of few samples [20], which has a certain contribution to plant breeding.

## Challenges

At present, the theoretical research of few-shot learning is in the stage of rapid development, and the applications of smart agriculture has just begun. Therefore, there are still many challenges. (i) Most of the existing crop and pest datasets are man-made rather than collected in the natural environment, which leads to the lack of robustness of the trained model and cannot effectively identify the objects in the real scene. (ii) The images collected based on the natural environment will have uneven illumination due to lighting, weather and other reasons. Therefore, they cannot be used directly and need to undergo data preprocessing, resulting in an increase in the workload in the early stage. (iii) The current agricultural few-shot learning work is just only theoretical research. If we want to truly realize few-shot recognition, we have to rely on technologies such as Internet of things (IoT) and embedded developments, which is also the most difficult and critical step in using few-shot learning in smart agriculture. (iv) Due to the different growth environments of crops, the implementation of technology needs to be adjusted to local conditions, which further increases the difficulty of implementation. (v) At present, there are not many studies on the application of few-shot learning in agriculture and plants, especially in plant phenotype and breeding. (vi) Finally, it is the challenge of few-shot learning itself. After all, it is not a mature technology, and it cannot completely get rid of the demand for the number of samples at present.

## Conclusions

With the continuous development of artificial intelligence technology, deep learning research is becoming more and more precise and mature. However, the shortcomings of relying on massive amounts of data in training are also slowly exposed. In the field of smart agriculture, collecting a large number of samples needs to involve high cost, difficult access, and privacy issues so it is very necessary to use few-shot learning. Few-shot learning has been emerging in recent years as an important branch, and the algorithms are mainly divided into four categories: based on data augmentation, metric learning, external memory, and parameter optimization. Moreover, using few-shot learning can also reduce the increasing burden of computer operation and save human and material resources for collecting samples. Since more advanced algorithms are being proposed in smart agriculture, they can assist farmers in monitoring crop growth, identifying pests and diseases, assisting plant breeding and many others.

## Data Availability

For relevant data and codes, please contact the corresponding author.
